# Risk of Mucormycosis in Diabetes Mellitus: A Systematic Review

**DOI:** 10.7759/cureus.18827

**Published:** 2021-10-16

**Authors:** Manish Khanna, Sabitha Challa, Ahmed S Kabeil, Bithaiah Inyang, Faisal J Gondal, Godwin A Abah, Mahesh Minnal Dhandapani, Manasa Manne, Lubna Mohammed

**Affiliations:** 1 Internal Medicine/Family Medicine, California Institute of Behavioral Neurosciences & Psychology, Fairfield, USA; 2 Medicine, Sri Ramachandra Institute of Higher Education and Research, Chennai, IND; 3 Research, California Institute of Behavioral Neurosciences & Psychology, Fairfield, USA; 4 Pathology, California Institute of Behavioral Neurosciences & Psychology, Fairfield, USA; 5 Internal Medicine, California Institute of Behavioral Neurosciences & Psychology, Fairfield, USA; 6 Internal Medicine, Federal Teaching Hospital Gombe, Gombe, NGA

**Keywords:** management of mucormycosis, diabetes mellitus and immunity, black fungus, mucormycosis, diabetes mellitus

## Abstract

Hyperglycemia or diabetes mellitus (DM) is a disorder of the endocrine system. In this condition, the body is insulin-deficient or resistant to insulin. Due to insulin deficiency or resistance, the body is unable to process sugar. The worldwide prevalence of diabetes mellitus is rising substantially.

Hyperglycemia makes the immune system weak, which increases the risk of infection in a diabetic patient. Fungal infection is more common in DM. Mucormycosis is a rare fungal infection in a healthy individual, but in DM, it can cause severe complications and even be fatal if not treated adequately and timely. In our literature review, a total of 19 published articles from the PubMed database and Google Scholar were included. We combed the PubMed database and Google Scholar by using various inclusion and exclusion criteria. The result of the review study shows the increased risk of mucormycosis in a diabetic patient.

## Introduction and background

Diabetes mellitus (DM) is one of the most common endocrine diseases affecting millions of people worldwide. Cases of DM have risen by more than 300 million in the last 35 years. Its prevalence is increasing rapidly [1].

Diabetes mellitus is the leading cause of kidney dysfunction, blindness, strokes, lower limb amputation, and heart attacks. According to the World Health Organization (WHO), diabetes was the cause of 1.5 million deaths worldwide in 2019, making it the top 10 leading cause of death globally. Common symptoms of DM are frequent urination, increased thirst, hunger, fatigue, blurred vision, and numbness and tingling in the hands and feet [1]. Broadly, DM is divided into two categories, type 1 DM and type 2 DM [1].

Type 1 DM is due to the complete absence of insulin production in the body because of autoantibodies targeted against pancreatic beta cells [1]. In contrast, type 2 DM is due to resistance developed against insulin [1,2]. Insulin is an anabolic hormone produced by the islets of Langerhans in the pancreas. It helps in regulating blood sugar in the body by increasing the uptake of glucose by various cells [1,2]. The glucose taken by cells is either stored in glycogen by the liver or used for energy expenditure by cells. Therefore, deficiency or resistance to insulin causes increased glucose in the blood [1,2]. Persistently high levels of blood sugar cause damage to blood vessels, nerves, and various cell types in the body. Complications associated with DM are diabetic retinopathy, neuropathy, and nephropathy [1]. DM also impairs the ability of the human body to fight infections by weakening cellular immunity. It also increases the time frame of recovery from an infection or injury [1,2].

Common factors which help in preventing and delaying the onset of DM are regular physical activity, eating healthy, and maintaining average body weight. Routine screening, proper diet, participating in physical activity, and medications help treat and prevent DM complications [1].

Mucormycosis is also called a black fungus. Cases of mucormycosis are infrequent but can be disastrous in patients suffering from immunocompromised conditions such as uncontrolled DM, coronavirus disease 2019 (COVID-19), and hematological cancers [2].

Mucor and Rhizopus are the joint offending agents and spread their infection mainly through inhalation of spores, but different routes are traumatic inoculation and ingestion [3]. Its spores are present in the soil, decaying food, and in the nasal cavities of a healthy individual. It commonly affects the lungs, sinuses, eyes, and brain and affects other body organs such as the stomach, intestine, and skin [4]. When spores enter the lungs, it causes pulmonary infection; when introduced in nasal cavities, it can spread to the brain and cause rhino-orbital-cerebral mucormycosis. Extreme malnutrition is often associated with the gastrointestinal (GI) form of mucormycosis [4]. Trauma and the use of infected instruments or medical supplies predispose to cutaneous form. Its presentation depends on the location it infects; rhino-orbital-cerebral mucormycosis presents with facial pain and headache, and if not treated timely, it can often lead to visual loss [5]. The relationship between DM and fungal infection is widely known, but the association of diabetes with mucormycosis is rare and more commonly seen in India and European countries [5]. This systematic review will show the risk and consequences of mucormycosis associated with DM.

Methods

Using the PubMed and Google Scholar databases, an in-depth systematic review was performed as per Preferred Reporting Items for Systematic Reviews and Meta-Analyses (PRISMA) guidelines [6].

Data Source and Strategy

We used keywords and the Medical Subject Heading (MeSH) strategy to explore and maximize the number of articles from PubMed and Google Scholar.

Following keywords are used for the search in Google Scholar as shown in Table 1.

**Table 1:** Keywords used in Google Scholar.

Keywords	Studies found on Google Scholar
Diabetes	3,780,000
Diabetes mellitus	2,870,000
Hyperglycemia	830,000
Diabetes mellitus OR hyperglycemia	2,250,000
Mucormycosis	34,200
Diabetes mellitus and mucormycosis	10,200
Diabetes mellitus, hyperglycemia, and mucormycosis	3,370

We used the following MeSH strategy to search for relevant papers: (Diabetes OR Hyperglycemia OR high blood sugar OR "Diabetes Mellitus/cerebrospinal fluid"[Mesh]) AND ("Diabetes Mellitus/diagnosis"[Mesh] OR "Diabetes Mellitus/diet therapy"[Mesh] OR "Diabetes Mellitus/drug therapy"[Mesh] OR "Diabetes Mellitus/epidemiology"[Mesh] OR "Diabetes Mellitus/microbiology"[Mesh] OR "Diabetes Mellitus/mortality"[Mesh] OR "Diabetes Mellitus/physiopathology"[Mesh] OR "Diabetes Mellitus/prevention and control"[Mesh] ) AND Mucormycosis OR Rhizopus OR Mucor OR ("Mucormycosis/cerebrospinal fluid"[Mesh] OR "Mucormycosis/complications"[Mesh] OR "Mucormycosis/drug therapy"[Mesh] OR "Mucormycosis/epidemiology"[Mesh] OR "Mucormycosis/microbiology"[Mesh] OR "Mucormycosis/mortality"[Mesh] OR "Mucormycosis/physiopathology"[Mesh] OR "Mucormycosis/prevention and control"[Mesh] OR "Mucormycosis/therapy"[Mesh]).

Inclusion and Exclusion Criteria

The inclusion criteria include articles published in English in the last 10 years (2011-2021), focusing on all age groups. Study types are used without any restriction, and all kinds of articles like clinical trials, randomized controlled trials, systematic reviews, traditional reviews, and case reports were explored. Restrictions such as age, ethnicity, and demographics were not taken into consideration during the review process. The exclusion criteria included publications whose abstracts were unavailable, incomplete articles, unpublished articles, and articles published before 2011.

Screening and Quality/Bias Assessment

We assessed 35 studies for quality using standardized quality assessment tools. The following methods were used: (1) randomized controlled trials (RCT): Cochrane Risk Assessment tool; (2) systematic reviews: Assessment of Multiple Systematic Reviews (AMSTAR) checklist or PRISMA checklist; (3) non-RCT and observational studies: Newcastle-Ottawa Scale; (4) case reports: Joanna Briggs (JB) check tool; and (5) any research paper without properly defined methods section: Scale for the Assessment of Narrative Review Articles (SANRA) checklist.

Results

**Figure 1:**
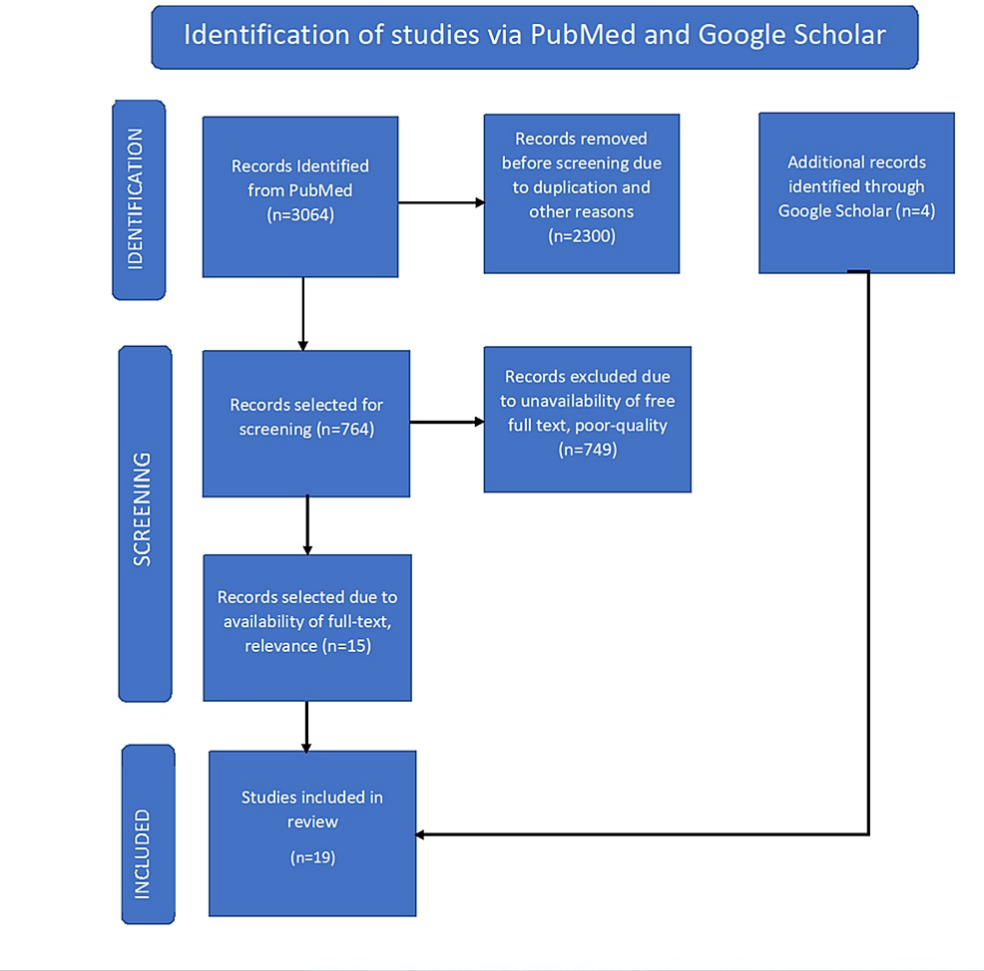
Preferred Reporting Items for Systematic Reviews and Meta-Analyses (PRISMA) flow diagram.

We identified articles using keywords and MeSH strategy for PubMed and Google Scholar databases. Using the MeSH strategy, we yield 3,064 articles from the PubMed database. Due to duplication and irrelevance, we excluded 2,300 articles from PubMed. We were left with 764 articles from PubMed before screening. We excluded 749 articles from the PubMed database based on inclusion-exclusion criteria, poor quality, and unavailability of free text. We selected 15 articles from the PubMed database based on free full text and relevance. To compensate for the excluded articles, we used the Google Scholar database. Keywords used for the Google Scholar database are diabetes mellitus, hyperglycemia, and mucormycosis. We selected four articles based on our inclusion-exclusion criteria from the Google Scholar database. Therefore, finally, 19 articles were selected for systematic review. PRISMA flow diagram is shown in Figure 1 [6].

## Review

Discussion

Pathophysiology of Diabetes Mellitus

In type 1 DM, autoantibodies are targeted against beta islets of Langerhans in the pancreas. The normal function of a beta cell is to produce insulin, which helps regulate blood glucose levels in the body [7]. An antibody is a molecule made up of protein and is a part-human immune system. The function of an antibody is to recognize and neutralize foreign molecules such as viruses, bacteria, parasites, fungi, and foreign antigens. When these antibodies cannot distinguish between self and foreign antigen and start attacking their own cells, they are termed autoantibodies. In type 1 DM, these antibodies attack the beta cells of Langerhans and destroy them. Due to the destruction of beta cells, the body is insulin deficient, and this leads to hyperglycemia [7]. Commonly found autoantibodies in type 1 DM are antibodies to glutamic acid decarboxylase (GAD-65), insulin autoantibodies (IAA), islet cell antibodies (ICA), and islet tyrosine phosphatase 2 antibodies (IA-2A) [7].

Type 2 DM is due to inadequate action of insulin and a decrease in the production of insulin by beta cells. Reduced sensitivity of cells to insulin leads to the development of insulin resistance [8]. To compensate for insulin resistance, the number of beta cells in the pancreas increases to produce more insulin [2]. Failure to do so results in type 2 DM. Obesity, aging, and physical inactivity also play a crucial role in developing insulin resistance in type 2 DM. Usually, obese patients with type 2 DM have more insulin in their blood, but they are insulin resistant than lean individuals with appropriate insulin sensitivity [2].

Diabetes and Immune System

Persistent high glucose levels in the blood interfere with the normal functioning of mitochondria and stimulate the production of a toxic molecule known as reactive oxygen species (ROS) [8]. The formation of ROS is harmful to multiple tissues and damages pancreatic beta cells and blood vessels due to oxidative stress. ROS causes mitochondrial dysfunction and destroys building blocks of the human body like proteins, nucleic acids, and lipids and therefore promotes aging [8]. For insulin production by pancreatic beta cells, cells require normal functioning mitochondria, but due to the damage caused by ROS, beta cells cannot synthesize a sufficient level of insulin [8]. As a result, it activates stress-responsive intracellular signaling molecules, which promotes cellular destruction. Diabetes accelerates the production of atherosclerotic plaques inside the blood vessels, leading to cardiovascular complications associated with it [8]. Increased blood glucose levels lead to covalent attachment of glucose and its toxic metabolites with protein, lipids, and nucleic acids. This process is known as non-enzymatic glycation, which results in advanced glycation end products (AGEs) [8]. AGEs damage tissues in many ways, such as blocking the signaling pathway of insulin, promoting inflammation by generating free radicals, and increasing the expression of pro-inflammatory cytokines, which results in diabetic complications of neuropathy, nephropathy, cardiomyopathy, and retinopathy [8].

Innate immunity and adaptive immunity comprise the immune system of the human body. Neutrophils, natural killer (NK) cells, and dendritic cells play a crucial role in innate immunity, whereas B lymphocytes and T lymphocytes are part of adaptive immunity [7]. DM impairs the ability of the human body to fight infection by interfering with innate and adaptive immunity [8]. Dendritic cells are antigen-presenting cells that present antigen to immune cells and helps them in the recognition and neutralization of infecting agents. Hyperglycemic patients are more susceptible to infection because of the reduced number of dendritic cells in circulation, increased apoptosis of NK cells, and neutrophilic dysfunction [7,8].

The immune system neutralizes Mucorales by chemotaxis, phagocytosis, and intracellular killing via the oxidative and non-oxidative mechanisms [9]. For the development of mucormycosis, germination of spores and the formation of hyphae are essential. In a healthy immunocompetent individual, all these functions are intact, but Diabetic patients are immunocompromised and lack normal functioning of immune cells [8,9]. Therefore, diabetic patients are more susceptible to develop mucormycosis [9,10].

Iron is a significant element in the development and cell growth of humans as well as infective agents. Therefore, pathogens utilize various methods for acquiring iron from the host [11,12]. Minimizing the availability of free or unbound iron is essential for preventing the proliferation of infective agents and against Mucorales specifically since it grows poorly without iron [11,12]. After the addition of iron, it multiplies. The availability of free iron and acidic pH in uncontrolled diabetes makes diabetic patients more prone to mucormycosis [13,14].

Diagnostic Criteria for Diabetes Mellitus

Diabetes can be diagnosed by various methods such as hemoglobin A1C (A1C), fasting plasma glucose (FPG), and oral glucose tolerance test (OGTT) [14].

We measure the average blood sugar for the past two to three months using the hemoglobin A1C test. Hemoglobin A1C level in the blood and its interpretation is shown in Table 2.

**Table 2:** Hemoglobin A1C values and their interpretation.

A1C value	Interpretation
Less than 5.7%	Normal
5.7% to 6.4%	Prediabetes
More than or equal to 6.5%	Diabetes

As the name suggests, in the fasting plasma glucose test, we check blood glucose levels after being fasted for at least eight hours before the test. You cannot eat or drink anything but water for at least eight hours beforehand [14]. Fasting plasma glucose levels and their interpretation are shown in Table 3.

**Table 3:** Fasting plasma glucose levels and their interpretation. FPG, fasting plasma glucose.

FPG level	Interpretation
Less than 100 mg/dl	Normal
100 mg/dl to 125 mg/dl	Prediabetes
More than or equal to 126 mg/dl	Diabetes

In the oral glucose tolerance test, we check blood glucose fasting after two hours of drinking a 75 g of glucose drink. It tells you about how well your body process glucose. OGTT is a more sensitive test than A1C and FPG [14]. The oral glucose tolerance test levels and their interpretations are shown in Table 4.

**Table 4:** Oral glucose tolerance test and its interpretation.

Blood glucose level after two hours of drink	Interpretation
Less than 140 mg/dl	Normal
140 mg/dl to 199 mg/dl	Prediabetes
More than or equal to 200 mg/dl	Diabetes

Mucormycosis

A fungal infection caused by a group of molds called Mucoromycetes is known as mucormycosis and is a rare but serious fungal infection. Spores of mucormycosis are present almost everywhere in nature, such as soil and decaying organic matter [9]. The introduction of its spores into the human body can be through inhalation, ingestion, and inoculation [9]. Commonly found agents which can cause infection in humans are Mucor species, Rhizopus species, Apophysomyces species, Rhizomucor species, Lichtheimia species, Cunninghamella bertholletiae, and Syncephalastrum species [15].

Mucormycosis can manifest in various ways in humans, depending on the location it infects. It can present as pulmonary, cutaneous, disseminated, gastrointestinal, and rhino-orbital-cerebral mucormycosis [16,17].

Rhino-orbital-cerebral mucormycosis is an infection that involves sinuses, orbits, and the brain. It usually starts by infecting sinuses and finally affects the eyes and the brain if not intervene promptly. It is most common in patients with uncontrolled DM and kidney transplant patients [18]. A most common complication of rhino-orbital-cerebral mucormycosis is blindness. Symptoms are facial pain and swelling, headache, and black-colored lesions in and around the oral cavity. The magnetic resonance and computed tomography of the paranasal sinus are the diagnostic techniques used in identifying the extent of the damage [18].

In pulmonary mucormycosis, lungs are infected by inhalation of spores. Cancer and organ transplant patients are at increased risk for pulmonary mucormycosis. Patients suffering from pulmonary mucormycosis have pneumonia-like systems such as shortness of breath, fever, and chest pain [18]. On imaging, it can present as consolidation, infiltration, and pleural effusion. Multiple nodules, thick-walled cavities, and lymphadenopathy are also seen in some patients [16]. These are nonspecific findings that make it hard to diagnose pulmonary mucormycosis [18].

Cutaneous mucormycosis is an infection that involves the skin. When the integrity of the skin is damaged, it allows the fungus to enter inside. Skin integrity is altered in a burn patient. Surgery, cut, and other skin traumas also harm skin integrity. In cutaneous mucormycosis, the patient had blisters or ulcers along with pain, redness, and swelling around it [18].

When Mucorales enter the blood circulation and spread to the various body parts, it is termed disseminated mucormycosis. If it extends to the brain, patients have altered mental status and coma. Patients with significant immunosuppression and profound iron overload are at risk of developing a disseminated infection [18].

Gastrointestinal mucormycosis presents as abdominal pain and GI bleeding. Its risk is increased in infants who are premature and have low birth weight if they had surgery, medication, and antibiotics since it weakens the immune system. It can also present in adults if they have diabetes mellitus or undergoing peritoneal dialysis [18].

Management of Mucormycosis

Early diagnosis and rapid management through various methods such as antifungal therapy, surgical intervention, and the use of hyperbaric oxygen play a crucial role in the management of mucormycosis. Rapid intervention helps in reducing morbidity and mortality associated with mucormycosis significantly [19,20].

Surgical debridement of necrotic or infected tissue greatly reduces mortality [21,22]. Parenteral use of antifungal agents such as amphotericin B, azoles, and caspofungin increase the survival rate in patients, and these agents can be used concurrently with surgical debridement [23,24]. Lipid formulation of amphotericin B is safer, less nephrotoxic, and efficacious as compared to amphotericin B [25]. In diabetic patients with mucormycosis, hyperbaric oxygen therapy is another alternative that increases the survival rate. Hyperbaric oxygen kills fungus by suppressing the growth of Mucorales, promoting neutrophil activity, stimulate the release of growth factors that help in wound healing, and increase oxygen delivery to necrotic tissues [26].

Combined and aggressive use of surgical debridement, antifungal therapy, and hyperbaric oxygen can significantly and drastically improve the outcome in patients suffering from mucormycosis [26,27].

The findings of the studies included in this review regarding the association of mucormycosis in diabetes patients are summarized below in Table 5.

**Table 5:** Studies showing the risk of mucormycosis in diabetes. COVID-19, coronavirus disease 2019.

Author	Year	Findings
Randhawa et al. [27]	2021	Development of invasive mucormycosis in a patient with a short course of dexamethasone.
Mishra et al. [5]	2021	Increased risk of mucormycosis seen in COVID-19 pandemic because of diabetes and corticosteroid use.
Martínez-Herrera et al. [24]	2020	Importance of identifying mucormycosis species for treatment purposes.
Ribes et al. [18]	2020	Diabetes mellitus is commonly associated with the different organisms of class Zygomycetes.
Mtibaa et al. [15]	2020	Prompt treatment is important for successful outcomes in mucormycosis.
Manji et al. [17]	2019	Atypical presentation of mucormycosis as angioinvasive fungal infection.
Serris et al. [4]	2019	Rhino-orbital-cerebral mucormycosis is more common in uncontrolled diabetes.
Barcenilla et al. [22]	2019	Increased risk of infection in diabetics is due to impaired cellular immunity.
Yeo et al. [21]	2018	Mucormycosis developed after functional endoscopic sinus surgery in a patient with a history of diabetes mellitus.
Raizada et al. [13]	2018	Ketoacidosis is a predictor of mortality in invasive fungal sinusitis with diabetes.
Corzo-León et al. [23]	2018	The most frequent form of mucormycosis seen in diabetes mellitus is rhino-orbital-cerebral mucormycosis.
Sahota et al. [22]	2017	Timely management and diagnosis are significant in survival and minimizing morbidity in rhinocerebral mucormycosis.
Mengji et al. [9]	2016	Mucormycosis is common in diabetes mellitus.
Jiang et al. [26]	2016	Diabetes mellitus is a common risk factor for invasive rhino-orbital-cerebral mucormycosis, which can initially present as orbital apex syndrome.
Kermani et al. [20]	2016	Rhinocerebral form of mucormycosis is most common and should be kept in mind in an immunodeficient patient.
Panigrahi et al. [11]	2014	Mucormycosis can be suspected as a cause of unresolved pneumonia in diabetic patients.
Singh et al. [19]	2013	High suspicion for rhinocerebral mucormycosis is important in diabetic patients who present with facial pain and swelling.
Berlanga-Acosta et al. [8]	2013	Hyperglycemia impairs the immune system that predisposes individuals to infection.
Khatiwada et al. [25]	2012	Different manifestations of mucormycosis in diabetes.

Limitations

This study has its limitation due to the minimal number of articles published exclusively illustrating the direct relationship between mucormycosis and DM.

## Conclusions

The impact of diabetes mellitus on the quality of life of millions of individuals is significant. Susceptibility or increased risk of infection in DM is due to poor immune response and readily available free iron in circulation, which is much more aggravated by high plasma glucose levels and acidic pH. Severe consequences of recurrent as well as rare infections are highly noticeable. One such rare infection which can result in disastrous consequences is mucormycosis. Factors such as decreased number of T lymphocytes, neutrophilic dysfunction, leukocytes apoptosis, and impaired dendritic cell function are the culprits behind severe complications associated with mucormycosis in DM. Earlier research shows that DM patients are more susceptible to severe complications related to mucormycosis than healthy individuals. Thus, there is a need to increase awareness regarding mucormycosis infection and its impact on health in the community to reduce the burden of disease.
